# [Retracted] MicroRNA-92b promotes tumor growth and activation of NF-κB signaling via regulation of NLK in oral squamous cell carcinoma

**DOI:** 10.3892/or.2025.9019

**Published:** 2025-11-11

**Authors:** Zhiming Liu, Cynthia Diep, Tiantian Mao, Li Huang, Robert Merrill, Zhouwen Zhang, Youjian Peng

Oncol Rep 34: 2961–2968, 2015; DOI: 10.3892/or.2015.4323

Following the publication of this paper, it was drawn to the Editor's attention by a concerned reader that the colony formation assay data shown in Fig. 4B were remarkably similar to the data shown in Fig. 2B for the CAL-27 experiments, albeit the panels in the latter figure were shown rotated through 90/180° relative to the former figure. Moreover, upon performing an independent analysis of the data in the Editorial Office, it came to light that some of the flow cytometric data in Fig. 2C were strikingly similar to data that had appeared previously in an article in the journal *Oncology Letters* that was written by different authors at different research institutes.

Given that the contentious data in the above paper had apparently already been published in an unrelated article prior to its submission to *Oncology Reports*, the Editor has decided that this paper should be retracted from the Journal on account of a lack of confidence in the presented data. The authors were asked for an explanation to account for these concerns, but the Editorial Office did not receive a reply. The Editor apologizes to the readership for any inconvenience caused.

